# VARIATION IN BIOLOGICAL RISK AMONG OLDER COLOMBIANS BY AGE, GENDER, AND EDUCATIONAL ATTAINMENT

**DOI:** 10.1093/geroni/igz038.2903

**Published:** 2019-11-08

**Authors:** Jennifer A Ailshire, Margarita Osuna

**Affiliations:** University of Southern California, Los Angeles, California, United States

## Abstract

Recent rapid aging in Colombia raises questions about the health status of older adults, but there has been very little research in this population. This study examines variation in biological risk by age, gender, and educational attainment using data from the 2015 SABE-Colombia, a nationally representative survey of Colombians ages 60 and older. Levels of cholesterol (total, HDL, and LDL), triglycerides, glucose, and hemoglobin were measured from whole blood and clinical cut-points were used to determine high-risk on each indicator. The five metabolic indicators were summed to create a total risk score; 58% of older adults had a score of 1 or more. Those ages 80 and older and women had lower risk, as did those with at least primary education. These patterns were also observed for high-risk on hemoglobin (13% prevalence), an indicator of anemia. Most older Colombians have some biological risk, but this varies by key subgroups.

